# Mpox: Exploring Epidemiology, Disease Outcomes, and Preventative Vaccination Among People with HIV During the Ongoing Outbreaks

**DOI:** 10.3390/v17121526

**Published:** 2025-11-21

**Authors:** Chloe Orkin, Ralph Torgler, Rebecca Dawson, Ian W. Holloway, Christian Hoffmann

**Affiliations:** 1 SHARE Collaborative, Blizard Institute, Faculty of Medicine and Dentistry, Queen Mary University of London, London E1 4NS, UK; 2 Bavarian Nordic Berna GmbH, CH-3174 Thoerishaus, Switzerland; 3 Word Monster, One, St. Peter’s Road, Maidenhead SL6 7QU, UK; 4 UCLA School of Nursing, Los Angeles, CA 90095, USA; 5 ICH Study Center, 20146 Hamburg, Germany; hoffmann@ich-hamburg.de; 6 Department of Medicine II, University of Schleswig-Holstein, Campus Kiel, 24105 Kiel, Germany

**Keywords:** HIV, mpox, MPXV, MVA-BN, PWH

## Abstract

Human mpox, caused by the mpox virus, is a reemerging viral zoonosis that has gained global attention due to recent Clade IIb outbreaks outside of Africa, as well as ongoing Clade Ia and Ib outbreaks in the Democratic Republic of Congo (DRC) and surrounding regions. Since the start of these outbreaks in 2022, approximately 160,000 people have been affected across more than 100 countries. People with human immunodeficiency virus (HIV; hereafter referred to as PWH) have been disproportionately affected, accounting for approximately 50% of all cases. Mpox is typically a self-limiting illness causing smallpox-like symptoms lasting 2–4 weeks, which can cause significant pain and morbidity. People with uncontrolled or advanced HIV face an elevated risk of severe mpox, secondary complications, and worse outcomes. Vaccination with second- and third-generation vaccinia-based smallpox vaccines has emerged as an important tool in mpox prevention, alongside behavioural modification to mitigate risk. However, only the third-generation, live-attenuated, non-replicating vaccine, modified vaccinia Ankara (MVA-BN [Bavarian Nordic]), is approved for use in PWH. Real-world estimates suggest that two doses of MVA-BN administered as pre-exposure prophylaxis confers vaccine effectiveness in the range of 66–90%. Additionally, MVA-BN has been widely demonstrated to have an acceptable safety profile. This narrative review explores the changing epidemiology, clinical manifestations, and outcomes of mpox in PWH. We also summarise evidence from the Clade IIb outbreaks on the effectiveness and safety of MVA-BN among PWH. Despite progress in our understanding, knowledge gaps persist regarding vaccine performance in individuals with advanced immunosuppression. Furthermore, due to the emergent nature of outbreaks in the DRC and surrounding areas, limited information is available regarding implications for PWH in the context of Clade Ia and Ib. We aim to provide healthcare providers, community stakeholders, and researchers with a foundational understanding of mpox in PWH and the role of MVA-BN in mpox prevention among this group, while highlighting areas of uncertainty. These insights may be helpful in the planning of future research and to inform strategies for the prevention and management of mpox among PWH, particularly those with advanced or uncontrolled HIV.

## 1. Introduction

Mpox, caused by the mpox virus (MPXV), is a member of the *Orthopoxvirus* (OPXV) genus and comprises two main genetic strains: Clade I (formerly Congo Basin/Central African Clade) and Clade II (formerly West African Clade) [1,2,3]. Each clade consists of phylogenetically distinct subclades, including Clades IIa and IIb [3], as well as Clade Ia and the recently characterised Clade Ib [4,5]. Historically confined to Central and West Africa, mpox has gained renewed global attention following recent multi-country outbreaks and the identification of novel viral subclades [4,6]. These events have highlighted a notable shift in affected populations, with people with human immunodeficiency virus (HIV; PWH) representing a disproportionately large share of cases in recent years [7].

In this narrative review, we aimed to synthesise the most relevant historical and up-to-date peer-reviewed literature sourced via PubMed, alongside data from the World Health Organization’s (WHO) continual global mpox surveillance programme [7], to summarise current knowledge on the evolving epidemiology, clinical characteristics, and outcomes of mpox in PWH. We also evaluate available evidence on the safety and effectiveness of the third-generation, non-replicating modified vaccinia Ankara (MVA-BN) vaccine (Bavarian Nordic A/S, Kvitsgård, Denmark) in this group, identifying key trends and knowledge gaps that may inform future research and public health strategies to support PWH who are affected by mpox.

## 2. Natural History of Mpox and Its Changing Prevalence Among PWH

### 2.1. Early Emergence of Mpox and Globalisation of Outbreaks

The first documented case of human mpox occurred in 1970 in an infant in the Democratic Republic of Congo (DRC) with Clade Ia infection [8,9]. Over the next three decades, cases of Clade Ia continued to increase in Central Africa, while Clade IIa emerged across Central and West Africa from 2000 onwards, establishing the virus as endemic in both regions [10,11,12]. In 2017, Nigeria reported its first outbreak in 40 years, with what is now recognised as Clade IIb [13].

Until 2022, cases of mpox outside of Africa were rare [11,14]. However, a surge in Clade IIb cases across all six WHO regions prompted the declaration of mpox as a Public Health Emergency of International Concern (PHEIC) from July 2022–May 2023 [15]. In August 2024, a second PHEIC was declared [6], due to ongoing, multi-country outbreaks of Clade IIb, in addition to outbreaks of Clade Ia and a new Clade Ib strain in the DRC and surrounding regions [4,5]. While the second PHEIC has very recently been lifted, mpox remains a persistent global public health challenge, especially for PWH [7]. Clade IIb continues to circulate globally at lower but persistent levels, having accounted for approximately 75% of all cases from 2022 until present day, though Africa is once again the most affected region due to increasing Clade Ia and Ib outbreaks [7]. As of October 2025, approximately 160,000 confirmed mpox cases and around 360 deaths have been reported worldwide since 2022 alone [7].

### 2.2. Evolution of Case Demographics

Historically, mpox primarily affected children and their predominantly female carers in endemic regions in Africa [16]. By the 2010s, the median age rose to around 21 years, coinciding with the emergence of Clade IIa and the first outbreak of IIb in Nigeria, during which adult males were predominantly affected [12,17]. Studies from the DRC in the 1990s suggested that HIV was not a significant co-factor in mpox prevalence at that time, although under-reporting of HIV status may have limited these findings [18,19]. Notably, 23% of the cohort studied during the 2017 Nigerian outbreak of Clade IIb were PWH, suggesting a potential association between HIV and increased mpox prevalence that may have been under-recognised at the time, though the overall sample size was small [17].

During the 2022–2024 multi-country Clade IIb outbreaks, the median age of infected individuals rose to 34 years, with 97% of cases occurring in males and 89% among gay, bisexual, or other men who have sex with men (GBMSM) [7,20]. Around half of all cases occurred in PWH, underscoring the stark change in affected populations compared with the earliest outbreaks, while also reflecting the continuation of patterns first observed in Nigeria in 2017 [7,17].

Regarding the ongoing Clade I outbreaks in Africa, recent data indicate that in previously unaffected regions, cases are mostly occurring in adults with an even sex distribution [7]. In historically endemic West and Central African regions, children under 15 years continue to be the most affected group [7]. At present, there is no definitive evidence of increased Clade I prevalence in PWH. Among 447 cases of Clade Ib in Kenya from July 2024–June 2025, approximately 23% of cases occurred in PWH, suggesting that PWH may have been disproportionately affected during these outbreaks [21]. Notably, however, the absolute case numbers were small, limiting interpretation of these findings. In any case, the emergent nature of Clade I outbreaks, limited surveillance capacity, limited laboratory capacity for HIV testing, and underreporting of HIV status in many affected countries hinder our current understanding.

### 2.3. Transmission Modalities and Implications for Risk

Early mpox cases were linked to zoonotic exposure, though by the 1990s–2010s, dynamics shifted towards human-to-human transmission via close physical contact [8,9,12]. Transmission during sexual contact was first described during Nigeria’s 2017 Clade IIb outbreak [17], but it was not widely accepted as the primary mode of transmission until it was reported again during the 2022 Clade IIb epidemic, accounting for approximately 85% of cases [7,22]. In particular, elevated Clade IIb risk is associated with the sexual networks of sexually active GBMSM, who remain the most affected group [23,24]. Given the higher global HIV prevalence among GBMSM compared with the general population (~7.7% vs. 0.7%), overlap with Clade IIb is expected, and is likely to reflect overlapping social and sexual networks rather than a direct biological link [25].

Current Clade Ib outbreaks in the DRC and surrounding regions show substantial evidence of transmission through sexual and non-sexual contact, raising concern for children and adolescents, as well as sexual networks [4,26,27]. An isolated case of Clade Ia in the DRC in a female sex worker also suggested possible sexual transmission [28], although zoonotic and close non-sexual contact are thought to be driving the current spread of Clade Ia in Africa, once again placing children and adolescents at elevated risk for this particular clade.

The initial isolated cases of Clade I that occurred outside of Africa from 2024 onwards were primarily linked to recent travel, with no evidence of onward transmission, though secondary household spread from a travel-acquired Clade Ib case was reported in a case in Germany [29]. However, in March 2025, the UK reported its first case of non-travel-related Clade Ib infection in an adult male [30]. Little is known about further onward spread from this particular case [30]. Since then, sporadic cases of locally acquired Clade Ib infection have been reported among men, including GBMSM, in Spain, the Netherlands, Italy, and Portugal [31]. Furthermore, in October 2025, three unrelated cases of Clade I infection (subclade unknown) were reported in California among GBMSM with no recent travel history and no known connection to one another [32]. Collectively, these reports indicate that recent Clade I cases outside of Africa are likely attributable to community transmission, potentially among overlapping sexual networks, though it remains unclear to what extent this modality may contribute to the further dissemination of Clade I outside of Africa. Should sexual contact continue to emerge as a dominant route of Clade Ib, or indeed Ia, transmission, then global Clade I risk may need to be reassessed, particularly for PWH.

### 2.4. Clade-Specific Case Fatality Rates (CFRs)

Historically, Clade I infections have been associated with higher case fatality rates (CFR) than Clade II, with estimates reaching up to 11% and 4%, respectively [12]. However, early estimates of Clade I CFR are likely inflated due to inaccurate reporting of confirmed cases, inadequate surveillance capacity, and limited healthcare, typical of that time period. Another factor to consider is the higher prevalence of Clade I infections in children and adolescents, who are known to be vulnerable to more severe mpox infections [12,33]. Thus, elevated CFR associated with Clade I may in fact be partly due to age-related vulnerability, as opposed to clade-specific differences in virulence, though social determinants, access to healthcare, immune status, and prior vaccination status are also important considerations [34]. In any case, 2024 estimates of Clade I CFR in the DRC suggest more conservative estimates of 0.2% in regions where both Clades Ia and Ib are currently circulating, indicating that historical Clade I CFR may have been overestimated [7]. By comparison, the global CFR during the 2022 multi-country outbreaks of Clade IIb was estimated at 0.18%, though this figure is not reflective of the heightened vulnerability of subpopulations, such as PWH who are immunosuppressed.

## 3. Clinical Features of Mpox Among PWH

Historically, the natural disease course of mpox was described as an initial, asymptomatic incubation period of 7–21 days, followed by a prodromal phase of non-specific symptoms, such as fever, dysphagia and chills, lasting approximately 1–4 days [33,35,36]. This was followed by an eruptive phase lasting 14–28 days, with mucosal and cutaneous lesions primarily on the face, trunk, and limbs [36]. Almost half of all individuals in earlier outbreaks had more than 20 lesions, though for some, lesions could be in the thousands [36].

However, during the 2022 Clade IIb outbreaks, clinical presentation appeared markedly different [35,37,38,39]. Patients reported fewer systemic symptoms, fewer lesions, and lesions primarily in the genital, oral, and perianal skin and mucosa, indicative of sexual transmission [35,37,38,39]. Some cases lacked prior prodromal symptoms, and asymptomatic and pauci-symptomatic disease were also described [40,41], making diagnosis and subsequent risk mitigation more challenging. Rectal pain, proctitis, and pharyngitis and dysphagia due to severe oral lesions were among the most common secondary complications [35,39]. Evidence from these outbreaks indicates that people with well-controlled HIV (on antiretroviral therapy [ART], CD4^+^ > 350 cells/mm^3^, undetectable viral load) experience similar disease severity and outcomes as those without HIV [37,42,43,44,45,46]. In contrast, those with advanced or uncontrolled HIV experience worse mpox-related outcomes, a longer duration of illness, and increased mortality compared to those with well-controlled HIV or those without HIV [38,45,47,48,49,50].

One case series among 382 individuals with advanced HIV from 19 countries demonstrated the global implications of these findings during the 2022–2023 Clade IIb outbreaks [38]. Outcomes were significantly worse among individuals with CD4^+^ cell counts of <100 cells/mm^3^ (advanced HIV), versus those with CD4^+^ cell counts > 300 cells/mm^3^ [38]. The former group experienced up to three times as many lesions, prolonged rash duration, and higher rates of complications, such as bacterial infections and severe anorectal ulceration. Respiratory involvement occurred in up to one-third of those with CD4^+^ counts < 100 cells/mm^3^, and hospitalisation rates reached 31% in this group. All mpox-related deaths occurred in individuals with CD4^+^ counts < 200 cells/mm^3^, with almost 1 in 4 individuals with CD4^+^ counts < 100 cells/mm^3^ dying from mpox complications [38].

US surveillance data mirror these findings in that over 90% of the 38 mpox-related deaths between May 2022 and March 2023 occurred in people with advanced HIV, typically with CD4^+^ counts < 50 cells/mm^3^ [51]. Similar patterns were also observed among PWH during the 2017 Clade IIb outbreaks in Nigeria, reinforcing the association between advanced/uncontrolled HIV and mpox severity, regardless of geography [17].

Regarding long-term sequelae, a recent small case study of three individuals with confirmed MPXV infection and advanced HIV (CD4^+^ counts < 150 cells/mm^3^) reported prolonged mpox illness characterised by persistent lesions and secondary complications, such as sepsis, resulting in the death of one individual 202 days after initial infection, with postmortem findings revealing the presence of MPXV antigen in his skin and lung tissue [50].

Collectively, these findings underscore immune status, particularly CD4^+^ cell count, as a major determinant of mpox disease course and outcomes among PWH. Furthermore, they highlight the need for longitudinal follow-up of PWH after mpox infection, particularly among people who are immunosuppressed, in order to ensure integration into the most appropriate care pathways where needed, thus reducing the risk of long-term complications. Ongoing case data among those with advanced HIV are needed to continue to build a fuller picture of long-term mpox outcomes among this group.

Although case data on Clade Ia and Ib infection in PWH are currently limited, disease outcomes among immunosuppressed individuals will likely mirror those from the 2022 outbreaks, underscoring the critical need for HIV diagnosis, pre-exposure prophylaxis (PrEP) for HIV and access to mpox vaccines in high-risk populations.

## 4. Treatment of Mpox Among PWH

As discussed above, individuals with advanced or uncontrolled HIV face elevated risk of longer and more severe mpox, thus prompt initiation of ART is crucial [24]. However, initiating or restarting ART late in the mpox illness may increase the risk of immune reconstitution inflammatory syndrome, linked to high mortality [38]. While no antiviral therapies are currently approved for mpox, tecovirimat is available under the Centers for Disease Control and Prevention’s (CDC’s) Expanded Access Investigational New Drug protocol for use in immunosuppressed individuals, including those with CD4^+^ counts < 200 cells/mm^3^ [52]. However, recent clinical trials reported no significant benefit of tecovirimat for the reduction in mpox lesion duration [53,54], though further analyses of these data are ongoing [55,56]. Other experimental options like brincidofovir, cidofovir, and intravenous vaccinia immune globulin may be used in severe mpox cases, though caution should be exercised to avoid potential drug interactions [57]. Pain management and access to appropriate clinical services are also important considerations, particularly when treating immunosuppressed individuals.

## 5. Preventative Vaccination with MVA-BN

### 5.1. Development and Approval of MVA-BN

Following the first-generation, live, replicating vaccinia-based smallpox vaccine, three additional vaccinia-based vaccines have been developed in the last 20 years, primarily for smallpox preparedness, with extended approval for mpox prevention. These include the second-generation vaccine, ACAM2000 (licenced in the US, Australia and Japan) [58], and the third-generation vaccines, LC16m8 (licenced only in Japan) [59], and MVA-BN [60].

MVA-BN is licenced under the names JYNNEOS^®^ (US, Mexico, Switzerland and Singapore), Imvamune^®^ (Canada), and Imvanex^®^ (UK, and European Union [EU]) [60,61,62,63,64]. In the context of mpox, it is approved for individuals ≥ 18 years of age [60,61,62]. Approval was also recently extended in the EU to adolescents aged 12–17 years [65]; while the WHO supports emergency “off-label” use in pregnant people and children at high risk [24,66].

Unlike ACAM2000 and LC16m8, MVA-BN is non-replicating in humans. It was developed by attenuating the highly virulent chorioallantois vaccinia Ankara virus over more than 600 continuous passages in chicken embryo fibroblasts and was designed to mitigate safety concerns associated with live replicating viruses used in first- and second-generation smallpox vaccines [60,67]. As such, only MVA-BN is indicated for use in immunosuppressed individuals, including PWH [24].

### 5.2. Current Vaccine Recommendations Among PWH

MVA-BN is recommended as a two-dose PrEP for PWH at high risk of mpox, according to the European AIDS Clinical Society (EACS), Canada’s National Advisory Committee on Immunization, and the WHO [24,68,69,70,71]. This includes sex workers, those travelling for sex, and GBMSM with a recently diagnosed sexually transmitted infection, multiple sexual partners, or those who have sex at sex onsite venues and their sexual partners [24,68,69]. EACS also recommends MVA-BN PrEP among all HIV PrEP users under the aforementioned circumstances [69]. For post-exposure prophylaxis (PEP) in PWH, MVA-BN should ideally be administered within four days of exposure but may be used up to 14 days post-contact, as per recommendations for people without HIV [24,68,69,70]. In the event of vaccine shortages, EACS recommends prioritising PWH, particularly those living with advanced HIV [69].

Two 0.5 mL doses of MVA-BN should be administered subcutaneously 28 days apart [60,61,62]. Off-label intradermal administration may be authorised in extenuating circumstances, such as in the event of vaccine shortages [24,72], though it is not currently recommended for individuals with immunosuppression due to insufficient safety data.

### 5.3. MVA-BN Effectiveness in the General Population

MVA-BN has been investigated in almost 9000 individuals across 20 clinical trials, though none have directly measured vaccine efficacy [62,73,74,75]. Instead, we are reliant on real-world estimates of vaccine effectiveness (VE) [76], which vary due to a lack of standardisation in the measurement of exposure, heterogeneous study designs, low numbers of events, and possible selection bias, thus warranting careful interpretation.

Nevertheless, from May–October 2022 almost 1,000,000 MVA-BN doses were administered in the US outside of clinical trials [77]. Additionally, over 75,000 GBMSM were vaccinated with MVA-BN in the UK between July 2022–December 2023, resulting in vaccine coverage of 37% for one dose and 50% for two doses [78]. Estimates of VE for MVA-BN PrEP during the 2022 Clade IIb outbreaks range from 35 to 89% after one dose and from 66 to 90% for two doses, based on data from subcutaneous and intradermal administration [62,76,78,79]. However, Brousseau et al. found one-dose VE increased from 35% to 65% after adjusting for exposure risk, such as HIV status, calendar time, and age, highlighting the importance of accounting for these factors when estimating VE [80].

Estimates of VE for MVA-BN given as PEP are limited and inconsistent [76,81,82]. One study reported VE of 89% from a single dose of MVA-BN PEP [81], while a second study reported VE of 78% [82]. However, re-evaluation of data from the latter study using target trial emulation, used in real-world studies to mimic randomised control trials and reduce bias, gave estimates of VE at around 19% [82]. Furthermore, findings from a recent study, which have not yet been published, report VE of MVA-BN as PEP at approximately 16% [83]. Given the small evidence base and variability in findings, firm conclusions about the effectiveness of MVA-BN as PEP are difficult to ascertain. In any case, time from MPXV exposure is likely to be a critical determinant of VE of PEP, though accurately pinpointing the moment of exposure is inherently challenging in real-world practice.

While current estimates of VE of MVA-BN, particularly PrEP, suggest meaningful protection, the role of vaccination in the resolution of the 2022 outbreaks remains uncertain. Modelling studies suggest behavioural changes and infection-induced immunity among GBMSM played the most significant role in expediting the resolution of outbreaks, though vaccination likely helped to prevent resurgence [84,85]. These findings align with data from Clay et al., who reported that while vaccination likely was not responsible for the initial decline, the combination of behavioural modifications and vaccination prevented an estimated 84% of cases one year into the Clade IIb outbreaks, highlighting the benefits of vaccination as part of a complementary strategy to combat mpox [86].

Estimates of VE in the context of Clade I are limited, though preclinical studies in animals have demonstrated the efficacy of MVA-BN against Clade I MPXV [87,88], supporting the likely effectiveness of MVA-BN during current Clade I, as well as Clade IIb, outbreaks.

### 5.4. MVA-BN Effectiveness in PWH

As discussed above, estimates of VE of MVA-BN for the prevention of mpox among PWH should be interpreted cautiously due to the limitations of real-world data. Nevertheless, real-world estimates of VE among PWH suggest similar protection to the overall population when both doses of MVA-BN are received, at approximately 70–80% [89,90,91]. Estimates of VE for partial vaccination among PWH are more variable, at 28–86% [91,92]. However, a recent prospective study in over 1200 PWH reported a one-dose VE of 35%, compared with 84% in those without HIV, suggesting that lower-end estimates of VE associated with partial vaccination in PWH may be more accurate, thus reinforcing the importance of completing the two-dose regimen among this group [93].

Notably, most studies among PWH focus on individuals with well-controlled HIV [89,91] or they do not describe CD4^+^ cell counts or ART use among their HIV cohort [92], limiting their relevance for those living with advanced HIV. Indeed, while the study by Dalton et al. reported VE of 70% among PWH and included individuals with CD4^+^ counts < 200 cells/mm^3^, VE data was not stratified by immune status [90]. Existing data on MVA-BN administered as PEP in PWH are also limited due to relatively small cohorts and low numbers of events [81]. Thus, additional research is needed to evaluate VE in those living with advanced HIV and to better understand the potential utility of post-exposure vaccination strategies among PWH. However, given the likelihood of reduced or highly variable VE of MVA-BN delivered as PEP in this population, vaccine recommendations should continue to prioritise MVA-BN PrEP in PWH.

In the case of breakthrough infections, vaccination with MVA-BN is associated with milder disease and fewer complications, hospitalisations, or deaths [78,93,94], even among PWH [93,95]. Indeed, in a large retrospective analysis of 273 confirmed mpox cases, 114 of which occurred in GBMSM with HIV, vaccinated individuals had less fever (23% vs. 59%, *p* < 0.001), headaches/chills (29% vs. 54%, *p* < 0.001), and swollen lymph nodes (38% vs. 56%, *p* = 0.02), compared with unvaccinated individuals [96]. There were no significant differences in symptoms or severity scores between individuals who received complete, versus incomplete, vaccination, nor were there any differences between PWH, versus those without HIV. Notably, however, the median CD4^+^ cell count among participants with HIV was 723 cells/mm^3^, indicative of well-controlled HIV [96].

### 5.5. MVA-BN Immunogenicity in PWH

A precise correlate of protection has not yet been established for MVA-BN. Nevertheless, in clinical trials conducted prior to the current mpox outbreaks, MVA-BN was shown to elicit seroconversion rates and neutralising antibody (nAb) titres, which were comparable to traditional smallpox vaccines [73,97]. Subsequent real-world studies during the ongoing outbreaks have further demonstrated the immunogenicity of MVA-BN using various immunological assays [98,99,100,101].

In PWH, the immunogenicity of MVA-BN was also initially evaluated in clinical trials prior to the current outbreaks [102,103,104]. In a Phase I/II study by Greenberg et al., 91 PWH on stable ART with CD4^+^ counts ≥350 cells/mm^3^ were compared with 60 individuals without HIV [102]. Two weeks after the primary dose of MVA-BN, over 80% of individuals in either group achieved seroconversion, defined as seropositivity in those initially seronegative or a ≥2-fold increase in those with pre-existing antibodies [102]. Antibody kinetics were similar regardless of HIV status or prior smallpox vaccination, though smallpox vaccine-experienced individuals demonstrated stronger, booster-like responses [102].

These results were supported by a larger Phase II trial by Overton et al. involving 579 PWH with CD4^+^ counts of 200–750 cells/mm^3^ [103]. Over 80% of smallpox vaccine-naïve participants and ≥97% of vaccine-experienced participants mounted robust immune responses, regardless of HIV status [103]. Although GMTs were slightly lower in PWH, they remained comparable to those induced by first- and second-generation smallpox vaccines [97,102,103]. While inclusion criteria appeared to favour individuals with well-controlled HIV, subsequent unpublished analyses by Bavarian Nordic revealed that up to 40% of participants had a CD4^+^ nadir < 200 cells/mm^3^, up to 20% had previously dropped below 100 cells/mm^3^, and over one-third had experienced a prior AIDS-defining illness [105]. Thus, these findings may be more generalisable to individuals with advanced HIV than originally assumed, though more direct evidence is needed [105].

Overton et al. later explored MVA-BN dosing regimens in PWH with a history of AIDS (CD4^+^ nadir < 200 cells/mm^3^) [104]. They reported comparable immunogenicity between standard-dose (SD; one dose at Weeks 0 and 4) and double-dose (DD; two doses at Weeks 0 and 4) regimens up to 12 months after vaccination, consistent with previous observations [102,103]. Although the booster-dose regimen (BD; SD regimen plus a booster at Week 14) induced higher peak nAb titres than the SD (281.1 vs. 78.9), SD titres remained within the range previously observed with ACAM2000 in healthy individuals, while the booster effect echoed earlier findings in vaccine-experienced participants [73,104].

Recent real-world studies reinforce these earlier findings. In a study that included 17 PWH, Guner et al. demonstrated robust OPXV-specific immunoglobulin G (IgG) responses following two doses of MVA-BN, reporting that the magnitude of response was greater among those with prior smallpox vaccination versus those without, including among PWH [106]. In the context of a single-dose regimen, Mazzotta et al. noted that smallpox vaccine-experienced PWH were less likely to seroconvert after a single dose of MVA-BN, compared to smallpox vaccine-naïve PWH who received two doses [107].

Together, these findings affirm the immunogenicity of MVA-BN in PWH and highlight the priming effect of prior smallpox vaccination. Furthermore, they collectively support the importance of completing a two-dose regimen in PWH to achieve a timely and robust immune response, regardless of prior smallpox vaccination [98,102,103,104,106,107]. Ongoing studies, including NCT05562323 (MoVIHvax cohort), are expected to provide further insights into humoral and cellular immune responses in PWH, including those with advanced or uncontrolled HIV.

### 5.6. Long-Term Protection and Booster Considerations in PWH

Understanding the durability of MVA-BN-induced immunity is essential for guiding booster policy, especially in PWH. While the exact duration of protection remains unknown, studies in healthcare workers in the DRC showed that OPXV-specific IgG responses peaked at Day 42 post-vaccination with MVA-BN but declined over time [98]. However, while IgG and nAb titres returned to around baseline levels two years post-vaccination, the majority of participants remained seropositive [98]. Subsequent studies confirm declining MPXV nAb levels within the first year after vaccination with MVA-BN, though low levels of MPXV nAb persisted up to one year later, particularly in smallpox vaccine-experienced individuals [108,109]. Conversely, robust MVA-BN-specific T-cell responses were observed at one year, regardless of prior vaccination or HIV status [108,109]. In the absence of a defined correlate of protection, the implications of waning antibody titres on long-term protection remain unclear. Additionally, natural mpox infection appears to induce more persistent humoral and cellular immunity than vaccination, leading to some public health agencies to suggest that vaccination may not be necessary in those with confirmed prior infection [110,111]. However, evidence remains relatively limited, and further studies are needed to determine whether natural infection provides sufficient and lasting immunity, particularly in PWH.

For those without prior infection, breakthrough case data can offer some indirect real-world insight into the durability of MVA-BN-induced protection, in the absence of established immune correlates. Among >32,000 mpox cases in the US from 2022 to 2024, <1% occurred in fully vaccinated individuals, with a median time to infection of 266 days after dose two [94]. However, wide variation in time to infection (14–621 days) suggests that exposure risk, not waning immunity over time, was the primary driver [94]. Similar findings were reported in the UK, where recipients of a two-dose regimen had slightly higher infection rates than single-dose recipients [78]. These findings likely reflect higher exposure risk rather than vaccine failure, underscoring the importance of reducing behavioural risk alongside vaccination, as well as highlighting the need to account for exposure patterns when interpreting the apparent duration of protection conferred by MVA-BN [78].

While the duration of protection conferred by MVA-BN remains uncertain, exploring the “boostability” of the vaccine is an important consideration. A Phase II follow-up study showed that a single MVA-BN booster given two years after primary vaccination induced a rapid and robust nAb response, exceeding peak levels observed with priming doses and remaining elevated at six months [74]. Unpublished interim results from a study in the DRC (NCT02977715) report similarly robust immune responses to a five-year booster, while pre-booster data appear to indicate that immunogenicity may persist for at least five years [112]. Data from a seven-year timepoint are being evaluated [112]. Although these data cannot be fully appraised until publication of the complete dataset, taken alongside previous findings, they appear to indicate that, should immunogenicity wane, booster doses are likely to be effective at restoring immune responses [74,112].

Currently, no global consensus exists on the need for MVA-BN boosters, nor their optimal timing. The CDC does not recommend boosters for people who received two doses of MVA-BN during the multi-country outbreaks of mpox, including PWH, though they advise two-yearly boosters for those at ongoing occupational risk [112]. In contrast, France recommends a booster ≥ 2 years after completion of the two-dose regimen in smallpox vaccine-naïve individuals who are not immunosuppressed, and in all immunosuppressed individuals regardless of prior smallpox vaccination [113]. Ongoing studies, such as NCT06885853 in France, aim to inform booster policies by evaluating long-term immunogenicity in high-risk groups, including GBMSM and users of HIV PrEP [113]; however, further evidence is needed to guide global booster recommendations, particularly for PWH who are immunosuppressed.

### 5.7. MVA-BN Safety Among PWH

The following section provides a targeted summary of the safety profile of MVA-BN in the general population and in PWH, highlighting the most clinically relevant safety considerations to guide clinical practice. More detailed safety analyses in the general population have previously been reported [60,93].

MVA-BN is widely considered to be well-tolerated with an acceptable safety profile among the general population [62,93]. Injection-site reactions and mild-to-moderate systemic reactions, such as headaches and nausea, are the most commonly reported adverse events (AEs) [62,93]. In pooled data from 23 studies with 8,988 participants aged 18–80 years, serious AEs occurred in 1.5% of MVA-BN recipients, versus 1.4% with placebo [60]. Cardiac adverse events of special interest occurred in 1.3% of vaccinated individuals and among 0.2% who received placebo, though fewer than 0.1% of these events were vaccine-related, and none were classed as serious [60].

Phase I/II trials in PWH report similar safety findings with no notable differences in AEs based on HIV status, even among those with a history of AIDS [102,103,104]. Furthermore, Overton et al. observed comparable safety with their SD and DD dosing regimens among PWH, although the occurrence of AEs was generally numerically higher with their BD regimen, consistent with expected booster reactogenicity [104]. Interestingly, earlier studies from both Greenberg and Overton involving individuals with well-controlled HIV suggested that MVA-BN may in fact be better tolerated in PWH compared to those without HIV, with fewer injection-site reactions in the former group [102,103]. A real-world study that included 1920 PWH aligned with these findings, reporting mainly mild-to-moderate AEs and reduced reactogenicity in PWH compared with those without HIV, though as with the aforementioned studies, most individuals in this cohort had well-controlled HIV [93].

Transient viral blips (VBs), which can occur in PWH on ART, are also an important safety consideration requiring evaluation to exclude treatment failure. Raccagni et al. studied the potential impact of MVA-BN on VBs (HIV RNA ≥ 50 copies/mL) and confirmed virologic failure (CVF; ≥1000 copies/mL or ≥2 readings ≥50 copies/mL) in 187 PWH post-vaccination [114]. Six VBs and three CVFs occurred over a median 1.97-month follow-up [114]. Those affected were older, had lived longer with their HIV diagnosis, had lower CD4^+^ counts, and prior viremia [114]. Among those experiencing VBs, the highest HIV-RNA loads were detected after a two-dose regimen, though VBs resolved after one month [114]. For the reported cases of CVF, a change in ART was needed [114]. It is unclear whether the incidence of VBs and CVF in this study exceeded expected occurrences in PWH on ART [114].

Regarding clinical considerations for vaccinating PWH, MVA-BN appears to be well-tolerated among this group. Mild injection-site and systemic reactions are common but generally mild in nature and self-limiting, aligning with the overall safety profile of MVA-BN. As discussed, transient VBs can occur in PWH, particularly in individuals with lower CD4^+^ counts or detectable HIV RNA, but these events are relatively uncommon and typically resolving, while CVF may also be reversed with a change in ART regimen. Nevertheless, caution is advised when considering a second dose in PWH with a detectable HIV-RNA load after receiving the first dose, especially in individuals living with advanced or uncontrolled HIV. Clinicians should monitor viral load post-vaccination among these individuals to minimise this risk. Overall, these data support the use of MVA-BN vaccination in PWH, though additional vigilance is recommended in higher-risk individuals. Ongoing monitoring and data collection in PWH should also be prioritised to ensure the continued safe and effective use of MVA-BN and to guide clinical decision-making, particularly among individuals who are immunosuppressed.

## 6. Discussion

The multi-country outbreaks of Clade IIb in 2022 prompted a new level of focus on mpox following a long period of global neglect, during which time mpox was largely confined to Africa. Despite the initial resolution of the first wave of Clade IIb outbreaks in 2022–2023, recent surveillance data suggest that Clade IIb continues to circulate globally at lower, but persistent, levels. Furthermore, Clade Ia and the newly emerging Clade Ib are rapidly spreading throughout the DRC and surrounding regions, with isolated cases of Clade I in at least 10 countries outside of Africa [7].

Unlike earlier mpox outbreaks, since 2022, PWH have accounted for almost half of all Clade IIb cases, and those with advanced or uncontrolled HIV have experienced higher mortality, longer duration of illness and poorer disease outcomes. Despite the current lack of case data from the ongoing Clade I situation in the DRC and beyond, poorer mpox-related outcomes among this population are anticipated, particularly in settings with limited access to HIV care. Hence, there is a clear need to examine the burden of mpox among PWH and to assess the role of vaccination in disease prevention, particularly in those who are immunosuppressed.

Alongside behavioural modification to alleviate risk, MVA-BN has shown promising real-world effectiveness for the prevention of mpox thus far, with immunogenicity data indicating a robust early immune response following one- or two-dose regimens. Perhaps not unexpectedly, prior smallpox vaccination results in a priming effect, increasing the magnitude of immune response. In PWH, VE and immunogenicity data indicate the likelihood of better protection from a two-dose regimen, irrespective of prior smallpox vaccination, with estimates of VE of MVA-BN PrEP comparable to observations among the overall population. Initial evidence also suggests a degree of sustained immunity for up to two years post-vaccination with MVA-BN, even in PWH. Furthermore, findings of a rapid and robust immune response following a booster dose of MVA-BN suggest that, should they be required, booster doses can elicit strong immune responses, which may be the key to ensuring lasting protection. Importantly, MVA-BN is considered to be well-tolerated in people with and without HIV.

### 6.1. Clinical Considerations for Vaccination Among PWH

Current guidance recommends a two-dose regimen of MVA-BN as PrEP for PWH. Evidence to support the utility of MVA-BN as PEP in PWH is currently lacking. However, if administered as PEP, standard guidelines for the general population should be followed, i.e., administration ideally within 4 days of exposure, and up to 14 days after exposure if necessary. Guidance on booster recommendations for PWH have not yet been universally established, and recommendations may evolve as WHO and other public health authorities continue to refine risk stratification and update global policy on mpox. Clinicians should remain aware of emerging guidance and consider individual patient risk factors when planning vaccination with MVA-BN. As discussed within, continual post-vaccination monitoring of immunosuppressed individuals is vital to minimise the risk of complications, such as transient VB or CVF, which may occur in immunosuppressed individuals, though occurrence is relatively rare.

Regarding overall patient care in PWH who have mpox, establishing effective HIV PrEP as early as possible should also be a priority, though due care and ongoing assessment should be exercised with any newly established or re-initiated ART regimen in light of the potential risk of immune reconstitution inflammatory syndrome.

### 6.2. Evolving Challenges and Critical Knowledge Gaps

Despite substantial progress in our understanding of mpox, particularly in the context of the current outbreaks, there are a number of uncertainties that must be addressed. Firstly, while existing vaccination data in PWH are promising, continual study in larger cohorts, including individuals with advanced or uncontrolled HIV, are vital to further our understanding. Secondly, though initial evidence suggests a degree of sustained immunity for up to two years post-vaccination, even in PWH, further research is needed to confirm the duration of protection conferred from MVA-BN and the subsequent requirements for booster doses.

We must also broaden our understanding of the implications of MVA-BN in children and adolescents with HIV, given the WHO’s recent approval of “off-label” prescribing of MVA-BN among these groups, as well as the fact that children remain at high risk of exposure for Clade I in the DRC and surrounding areas. Any studies in these cohorts should initially be performed among those with well-controlled HIV to minimise the potential risk or likelihood of adverse outcomes.

Crucially, future studies involving PWH must strive to ensure detailed and adequate reporting of clinical parameters such as HIV viral load, CD4^+^ cell counts, and ART use to allow for thorough interpretation of the data. Vaccine recommendations should also be continually evaluated and revised as necessary based on emerging evidence, particularly among vulnerable groups.

### 6.3. Future Research Priorities

While several critical evidence gaps remain, designing future studies to address them presents increasing challenges. The decline in Clade IIb incidence since its 2022 peak may limit opportunities to generate new VE data in high-income settings, where the majority of previous mpox research has taken place, due to the likelihood of limited number of incident cases of mpox. Conversely, ongoing Clade Ia and Ib outbreaks are mainly concentrated in sub-Saharan Africa, where limited surveillance capacity and research infrastructure in some regions may hinder research efforts. The WHO’s ongoing global mpox surveillance programme [7] provides an important infrastructure for data collection and case monitoring across multiple countries, including sub-Saharan Africa, but limitations in local research capacity and cohort follow-up mean that comprehensive VE or immunogenicity studies may still be difficult to implement. This may be particularly true of potential studies involving PWH, where underreporting of HIV status could further complicate study design and data interpretation.

Hence, future research efforts may need to rely more heavily on long-term real-world data collection, pooled multi-country observational cohorts, and enhanced genomic and immunologic surveillance, rather than traditional large prospective trials. Continued collaboration between global health agencies and local research networks will be vital to ensure equitable data generation and representation across settings. Although some real-world studies on booster duration are already underway, addressing these broader structural and logistical barriers will ultimately determine how effectively future research can fill current knowledge gaps.

### 6.4. Stakeholder and Public Health Considerations

In the meantime, it is crucial to continue to closely monitor the global mpox situation, as well as Clade I-specific dynamics in the DRC and neighbouring countries, to help guide timely and context-appropriate public health responses, particularly among PWH. HIV status should routinely be reported alongside mpox diagnosis, and routine HIV testing should be conducted in individuals with mpox, especially those experiencing severe mpox symptoms. The roll out of HIV PrEP to prevent new HIV infections is also vital and mpox care and vaccination settings should be leveraged to offer HIV PrEP where appropriate.

Above all, equitable access to testing and vaccine resources must remain a priority, particularly in the worst affected areas. At present, collaborative efforts from international agencies aim to allocate doses of MVA-BN to the highest-risk regions, yet substantial gaps remain, especially in resource-limited settings where HIV prevalence is high. In these environments, persistent structural barriers, such as inadequate vaccine storage and distribution infrastructure (cold-chain infrastructure), inconsistent vaccine supply chains, and limited integration between HIV and mpox vaccination services, continue to constrain equitable access to resources.

To assist in vaccine procurement, the WHO recently prequalified MVA-BN amidst the ongoing outbreaks, marking it as the first prequalified mpox vaccine [66]. Additionally, the WHO, the Africa CDC, the Coalition for Epidemic Preparedness Innovations, Gavi, and UNICEF are currently collaborating on an Access and Allocation Mechanism to procure mpox vaccine doses for those most at risk [115]. Their planned target was to reach 5.85 million pledged mpox vaccine doses by the end of 2024, with approximately one million doses already allocated to the nine worst-affected countries in Africa [115]. Over 2 million doses of MVA-BN alone have thus far been pledged from the EU, US, Canada, Gavi, and the MVA-BN manufacturer, Bavarian Nordic [115].

However, access to resources alone is unlikely to alleviate the current global situation. Political will is vital to ensure the correct allocation of funding and resources, to implement clear vaccine policies, to coordinate vaccination efforts, and to address gaps in education through public engagement. The latter strategy may be particularly important in reducing stigma associated with targeted vaccination efforts among PWH. Such efforts to address risks within affected communities must be done with care, avoiding assumptions based on HIV status, sexual orientation, or occupation.

### 6.5. Limitations of This Review

As this is a narrative review, and not a systematic review, its reproducibility is limited and the content could arguably be subject to risk of bias, as with all narrative reviews. Detailed case data on Clade Ia/Ib are also relatively sparse in comparison with Clade IIb, particularly in the context of PWH, meaning that discussions around these topics are partly speculative and hypothesis-driven. Our interpretation of VE, clinical outcomes in PWH, and epidemiological patterns is constrained by the availability of robust data, which are largely derived from Clade IIb cases outside of Africa, thus potentially limiting the generalisability of findings to other settings or populations. Furthermore, the rapidly evolving nature of the global epidemiology of mpox means that our current thinking is also likely to be continually challenged and extended as new data continue to emerge. Nevertheless, this narrative review provides a highly detailed contemporary synthesis of the existing literature pertaining to mpox in PWH thus far, covering multiple thematic areas and re-examining historical data in the context of our evolving understanding, utilising over 100 credible literature sources. Even as our understanding evolves, we anticipate that this review will provide a detailed foundation that can inform future research, clinical practice, and public health strategies.

### 6.6. Summary

In summary, the current mpox outbreaks highlight the need for a comprehensive approach to mpox management in PWH, such as early diagnosis; access to HIV PrEP; timely vaccination with MVA-BN; and most importantly, continual behavioural modification among those at high risk of exposure. Current safety and effectiveness data for MVA-BN among PWH suggest that vaccination may be a promising complementary strategy for mpox prevention. However, additional data are needed to better characterise outcomes in those with advanced or uncontrolled HIV, while future research is required to support use in children and adolescents with HIV. Although robust Clade Ia and Ib case data are currently lacking, the emerging outbreaks in Africa underscore the importance of continued vigilance, particularly among PWH who may face heightened risk. As the global community navigates this evolving health challenge, targeted interventions, equitable access to vaccines, and robust data collection will be critical to safeguarding the health of PWH and reducing the burden of mpox worldwide.

## List of Abbreviations

AE	Adverse event
ART	Antiretroviral therapy
BD	Booster dose
CDC	Centers for Disease Control and Prevention
CFR	Case fatality rate
CVF	Confirmed virologic failure
DD	Double dose
DRC	Democratic Republic of Congo
EACS	European AIDS Clinical Society
EU	European Union
GBMSM	Gay or bisexual men, or other men who have sex with men
GMT	Geometric mean titres
HIV	Human immunodeficiency virus
IgG	Immunoglobulin G
MPXV	Mpox virus
MVA-BN	Modified vaccinia Ankara vaccine
nAb	Neutralising antibody
OPXV	Orthopoxvirus
PEP	post-exposure prophylaxis
PHEIC	Public Health Emergency of International Concern
PrEP	Pre-exposure prophylaxis
PWH	People with human immunodeficiency virus
RNA	Ribonucleic acid
SD	Standard dose
UK	United Kingdom
US	United States
VB	Viral blip
VE	Vaccine effectiveness
WHO	World Health Organization
